# The relationship between L2 motivation and transformative engagement in academic reading among EAP learners: Implications for reading self-regulation

**DOI:** 10.3389/fpsyg.2022.944650

**Published:** 2022-09-29

**Authors:** Esmaeel Abdollahzadeh, Mohammad Amini Farsani, Maryam Zandi

**Affiliations:** Department of Foreign Languages, Iran University of Science and Technology, Tehran, Iran

**Keywords:** transformative experience, L2 motivational self-system, reading skill, disciplinary variations, L2 self

## Abstract

This study examined the relationship between L2 motivation and engagement in academic reading skill from the lenses of L2 motivational self-system and transformative experience. More specifically, following the transformative experience (TE) framework, we investigated the level of students’ engagement in academic reading skills inside and outside English classes. We also explored what motivational factors act as strong predictors of transformative experience and whether L2 motivation and engagement of students differ across different disciplines. Stratified purposive sampling was followed to recruit 419 undergraduate English for academic purposes (EAP) students studying in different majors. As such, we developed a questionnaire for measuring TE and utilized a pre-established questionnaire to operationalize L2 motivational self-system. We found that L2 motivation significantly covaried with students’ level of engagement in English academic reading skill. Furthermore, the results of multiple regression analysis revealed that L2 learning experience and ideal L2 self were strong predictors of transformative engagement; ought-to L2 self played a marginal role in the occurrence of TE. Discipline-wise, Life Sciences students were considerably more motivated than those in Arts and Humanities. However, no significant difference was observed in the extent of transformative engagement among students across disciplinary groups. Implications for EAP instructors, educational authorities, and material and curricula developers are discussed.

## Introduction

Second language researchers have been preoccupied with tracing students’ success in language learning. Besides different factors documented, it is argued that engagement is key to effective language learning (Zhang, 2020); i.e., it positively affects language awareness (Svalberg, 2009), language achievement, higher attendance in class, and intrinsic motivation to learn English (Dincer et al., 2017). In search for finding the paths to enhance students’ engagement with language, several studies examined the role different variables, such as learner-generated content (Lambert et al., 2017), online social networks (Akbari et al., 2016), and L2 motivations, have played in shaping and constructing engagement in language. More specifically, L2 motivation is closely intertwined with engagement (Fredricks et al., 2004; Yin, 2018). The literature highlights a significant and positive relationship between motivational factors, such as intrinsic motivation (Comanaru and Noels, 2009) and future self-guides (Asker, 2012), and engagement in language learning. This means engaged students are necessarily powered by some motivation that guides their activities (Dornyei, 2019b).

Engagement is a multidimensional construct with behavioral, affective, and cognitive facets (Fredricks et al., 2004). These dimensions are involved in learning L2 inside and outside class (Ben-Eliyahu et al., 2018). Fredricks (2011) argued that engagement caters for two facets: (1) what happens inside the class and (2) what happens beyond the class. Although a triple dimension of engagements has received great empirical attention in the literature (see Dincer et al., 2017), what is almost unaccounted for is engagement beyond the classroom. The gap becomes more evident when we explore engagement from the perspective of transformative experience. Transformative experience (TE), giving more weight to engagement out of the classroom, is a holistic framework of engagement with three qualities of motivated use, expansion of perception, and experiential value. It resonates with behavioral, cognitive, and affective dimensions (Pugh et al., 2017a).

TE construct has been originally developed within the domain of science education. One controversial issue there is why some students engage in TE while others do not (Pugh et al., 2019). In this regard, several studies explored the contribution of different factors (e.g., interest, personality traits, positive and negative emotions, anxiety, task values, parent involvement intervention). However, what is missing is lack of research in the domain of language learning. As Tang (2016) puts it, being fluent in a new language can be a transformative experience; therefore, transformative experience has the capability to extrapolate to L2 studies.

The literature highlighted the salient role L2 motivation can play in boosting students’ engagement in L2 classrooms (e.g., Dincer et al., 2017). A number of recent studies in the Asian contexts, such as Japan (Aubrey and Philpott, 2021), Indonesia (Subekti, 2018; Zansabil, 2019; Herwiana and Anam, 2022), Sri Lanka (Wijeratne, 2015), Pakistan (Rasool and Winke, 2019), China (Li and Liu, 2021), and Thailand (Swatevacharkul, 2021), have suggested the less significant role of ought-to L2 self, compared to other two factors of L2MSS, in motivating L2 learners. However, such considerable role has not been empirically examined for language skills, and notably, the association of L2 motivation with academic reading engagement both inside and outside in particular has remained underexplored.

Given the fact that motivation is fundamental for engagement in TE (in-class and out-of-class engagement; Alongi et al., 2016), the present study explored the role of L2 motivation in the transformative engagement of students in academic reading through the lens of L2 motivational self-system (L2MSS) to address the aforementioned shortcomings. The gap becomes more conspicuous when we examined this relationship between L2 motivation and academic reading engagement in English for academic purposes (EAP) contexts. Academic reading skill is the dominant language skill taught in academic EAP classroom in Iran. It varies from other kinds of reading in terms of the length and level of texts at different stages (Sohail, 2015). “Academic reading is a measured, challenging and multifaceted process in which students are dynamically engaged with a range of reading strategies” (ibid, p. 119). It has to do with asking questions while reading, establishing links to other reading texts, thinking about texts, comparing texts, interpreting meaning, and usually describing the reading again (Buterbaugh, 2021).

L2MSS is an L2 motivation framework, projected by Dornyei (2005), which consists of three subcomponents of “ideal L2 self,” “ought-to L2 self,” and “L2 learning experience.” To date, the majority of L2MSS research is being undertaken among EFL learners in general. The current study took a cross-disciplinary perspective and compared L2 motivation and its link with transformative engagement in academic reading skill among EAP learners. In this respect, applying Nesi and Gardner‘s (2006) classification scheme, we examined the interrelationship between L2 motivation and L2 engagement in EAP contexts with specific concentration on subject areas of “Arts and Humanities,” “Life Sciences,” “Physical Sciences,” and “Social Sciences.”

More specifically, the present study aims to primarily investigate the relationship between foreign language motivation and transformative engagement in academic reading skill among undergraduate EAP learners. We explored what subcomponents of L2 motivational self-system (L2MSS) act as the strongest predictors of transformative engagement in academic reading and whether L2 motivation and transformative engagement of university students vary across different disciplines. Another purpose of the current study was to compare the in-class academic engagement of university students with their out-of-class engagement.

A crucial concept in the current accounts of effective academic learning is self-regulated learning (Banarjee and Kumar, 2014), which is believed to be a significant element in academic success (Lindner and Harris, 1992) and even a favored educational outcome (Paris and Newman, 1990). It is generally acknowledged that there is a positive association between motivation and self-regulated learning (Al-Otaibi, 2013). Moreover, L2MSS is viewed as a self-regulation model (Mezei, 2008), and ideal L2 self (Al-Otaibi, 2013; Kim and Kim, 2014) and learning environment (Young, 2005; Alzubaidi et al., 2016) have been found to enhance self-regulated learning. The literature also suggests that engagement significantly contributes to self-regulated learning (Gaxiola-Romero et al., 2020; Estévez et al., 2021). However, the learners should be motivated to invest extra attempt and time (i.e., get more engaged) needed for self-regulated learning (Al-Otaibi, 2013). Therefore, it is hypothesized that the findings of his study regarding L2 motivation, TE, and the relationship between L2 motivation and TE in academic reading would contribute to self-regulation in academic reading skills.

The following research questions guided the study:

1) Is there any significant relationship between L2 motivation and transformative engagement in academic reading skill among undergraduate EAP learners?
2) What motivational factors are the strongest predictors of transformative engagement in EAP academic reading skill?
3) Are there any disciplinary discrepancies among undergraduate EAP learners in terms of the extent of transformative engagement in academic reading skill and L2 motivation?
4) Is there any significant difference between in-class and out-of-class engagement among undergraduate EAP learners?

### Literature review

#### Engagement

Engagement is characterized as “student’s psychological investment in and effort directed toward learning, understanding, or mastering knowledge, skills, or crafts that academic work is intended to promote” (Newman, 1992, p. 12). Engagement comprises behavioral, emotional/affective, and cognitive dimensions (Fredricks et al., 2004). “Behavioral engagement”—drawing on the idea of participation—includes involvement in academic and social/extracurricular activities. “Emotional engagement” involves positive and negative reactions to teachers, classmates, academics, and school. “Cognitive engagement” incorporates “thoughtfulness and willingness to exert the effort necessary to comprehend complex ideas and master difficult skills” (ibid, p. 60).

In L2 language learning, engagement has been investigated in both in-class and out-of-class contexts. As for the in-class engagement, Dincer et al. (2017) examined the relationship between the multidimensional classroom engagement (behavioral, emotional, cognitive, and agentic engagement) and a number of variables including course achievement, course absence, and motivational orientation to learn English. Their findings indicated that engagement in the classroom significantly contributed higher course achievement, higher attendance, and intrinsic motivation to learn English. Furthermore, Dincer et al. (2019), in another study with 412 EFL learners, explored the relationships between context (perceived autonomy support from the instructor), self (basic psychological needs), action (behavioral, emotional, agentic, and cognitive engagement), and outcome (achievement and absenteeism). The findings showed that achievement and absenteeism within English course were predicted by in-class engagement. The literature has also highlighted the importance of out-of-class engagement for language learning (Hyland, 2004; Inozu et al., 2010; Lai et al., 2015). For example, Inozu et al. (2010) investigated the relation of language-related activities outside class to language learning and personal development and found a positive relationship between them. This finding lends support to Nunan (1989) assertions that most of the students found classroom instruction itself to be insufficient for the development of English competence. Instead, they are striving for engagement in outside classroom learning which can improve their language development.

In addition to focusing on L2 learning in general, the role of engagement in developing second language skills, such as reading, has been highlighted in recent research. It is argued that engagement in reading can lead to reading achievement (Guthrie et al., 2012; Mahdikhani and Rezaei, 2015). For instance, Whitney and Bergin (2018) explored Grade 3 and Grade 5 teacher-rated classroom engagement as the predictor of reading achievement between three racial/ethnic groups (White, Black, and Hispanic) and five levels of socioeconomic status (SES). The results revealed that engagement positively predicted reading achievement. In this study, the White students in Grades 3 and 5 outperformed Black and Hispanic students in the reading test, and high-SES students, compared to low-SES students, achieved higher reading test score. Moreover, Barber and Klauda (2020) pointed out that engaged readers consider reading skill as a tool for enhancing their knowledge and strongly believe that reading is beneficial for their current and future life.

#### Transformative experience

Following Dewey’s (1934) writings on educational and aesthetic experience, Pugh (2002) projected transformative experience (TE) engagement construct. Transformative experience refers to “experiences in which students use concepts learned in school in their everyday experience to see and experience the world in meaningful and new ways” (Pugh et al., 2017a, p. 2). TE is composed of three components of motivated use, expansion of perception, and experiential value. Motivated use involves “the application of school content in contexts where application is not required, particularly in out-of-school contexts’ (Pugh et al., 2017b, p. 2). Expansion of perception refers to “seeing and understanding aspects of the world (objects, events, issues, others, or the self) in new ways” (Pugh et al., 2010a, pp. 3–4). Experiential value is defined as “the valuing of content for its usefulness in immediate, everyday experience” (ibid, p. 4). TE is a worthy learning outcome by itself (Pugh et al., 2010a). In addition, a number of studies indicated that students’ TE promotion supported enduring learning (Girod et al., 2010) and conceptual change (Alongi et al., 2016).

Heddy and Sinatra (2017) explored parental intervention as the facilitator of TE and suggested that engagement in TE is likely increased through a parent intervention. In a recent study, Pugh et al. (2019) investigated the role of interest, personal traits, emotions, and task values in the occurrence of TE. The findings suggested that interest could significantly predict TE. Moreover, it turned out that as task values (intrinsic value, utility value, and attainment value) and positive emotions fostered, TE increased. In reverse, personal traits, generally, did not make strong contribution to TE. Reyes and Johnston (2018) carried out a case study in a construction surveying course to investigate the role of motivation in the enhancement of the transformative experience. In this study, they used gamification, as a motivating tool, to increase the student’s motivation and in turn their transformative experience. The result revealed that as the motivation of the students increased, they became more engaged in the transformative experience.

#### L2 motivational self-system

In this study, L2 motivational self-system (L2MSS), developed by Dornyei (2005), was followed as the theoretical framework to trace students’ L2 motivation. Dornyei (2005), synthesizing two theoretical motivation constructs of Noels (2003) and Ushioda (2001) and using his findings, proposed his L2 motivational self-system. L2MSS contains three subcomponents, two of which are future self-guides and pertain to learners‘ possible selves: ideal L2 self, ought-to L2 self, and L2 learning experience. Ideal L2 self refers to “the representation of the attributes that someone would ideally like to possess (i.e., a representation of personal hopes, aspirations or wishes)” (Dornyei and Ushioda, 2009, pp. 3–4). Ought-to L2 self involves “the attributes that one believes one ought to possess to meet expectations and to avoid possible negative outcomes (ibid., p.29).” The L2 learning experience is related to “executive” motives related to the immediate learning environment and experience [e.g., the impact of the teacher, the curriculum, the peer group, the experience of success (ibid., p. 29)]. We adhered to this model as it has been tested and validated in different EFL contexts (e.g., Teimouri, 2017; Doiz and Lasagabaster, 2018; Yetkin and Ekin, 2018). It is further the latest L2 motivation model represented in the motivation literature (see Huhtala et al., 2019).

#### L2 motivational self-system and transformative experience

Pugh (2004) and Pugh et al. (2010a) stated that students’ identity plays a significant part in developing TE. Pugh et al. (2010a) investigated the role of identity, in particular science identity, as the predictor of TE. In defining identity, they referred to the concept of possible selves and imagination. Those students who could imagine themselves perusing the study or work related to their field of study reflected their engagement in TE (see Girod and Wong, 2002; Pugh, 2004). The result confirmed the important part of identity (including possible selves) in the occurrence of TE.

As can be seen, possible selves and imagination, which are the key elements and concepts of L2MSS, positively contribute to the occurrence of TE.

Another association of the L2MSS with TE relates to the L2 learning experience (third component of L2MSS). This component is broken down into the facets, such as school context, syllabus and teaching materials, learning tasks, one‘s peer, and teacher (Dornyei, 2019a). In this regard, Crawford (2018) implemented TE pedagogy on the students of different disciplines. The result revealed that peer-to-peer and group works led to a notable enhancement in the TE of the students. Besides, there is consensus that TE of students is influenced by teachers (Girod et al., 2010; Pugh et al., 2010b; Clifford and Montgomery, 2015; Crawford, 2018). Accordingly, it can be presumed that facets of L2 learning experience can positively contribute to TE.

The majority of L2MSS-based studies have undertaken among EFL learners in general; few cross-disciplinary studies have been conducted through the lens of L2MSS. In this respect, Martinović (2018), adhering to L2MSS framework, examined the English language learning motivation of the students who were studying in different disciplines, including Biomedical and Health Sciences, Biotechnical Sciences, Humanities, Social Sciences, and Technical Sciences. The findings revealed that the biotechnical students had the weakest ideal L2 self and ought-to L2 self, compared to the other disciplines. Hosseini and Shokrpour (2019) explored the motivating factors that affected medical and nursing ESP learners. The result indicated that medical students were more motivated than nursing ones for learning English. Although both ideal L2 self and L2 learning experience were revealed as important motivating factors, both medical and nursing students found the latter one a more powerful predictor of their motivation. Given Pugh’s framework of transformative experience (2002), it seems that there is a lack of cross-disciplinary investigation on engagement through the lens of this framework, notably in the field of language learning.

#### Motivation and self-regulated learning

Self-regulated learning is often described from the perspective of motivation and learning strategies (Fokkens-Bruinsma et al., 2020). Some studies have used the more comprehensive concept “self-regulation” instead of language learning strategies, due to the measurement challenge and definitional fussiness (Dornyei, 2005; Tseng et al., 2006; Rose, 2012; Banisaeid and Huang, 2014; as cited in Banisaeid and Huang, 2015). According to Oxford (1990), the learners with a higher level of motivation adopt a considerably wider range of suitable strategies than do the less motivated learners. Moreover, many studies have found a positive relationship between motivation and self-regulated learning (e.g., Mahmoodi et al., 2014; Banisaeid and Huang, 2015). Interestingly, L2MSS can be regarded as a self-regulation model, because “it can explain motivation in terms of goals (the ideal self), monitoring (the discrepancy between the actual and the ideal self), and choices (reactions, decisions as to how to refine goals, and planning)” (Mezei, 2008, p. 81), which are seen as the self-regulation stages (Pintrich, 2000, as cited in Mezei, 2008). It is also reported that the ideal L2 self (Al-Otaibi, 2013; Kim and Kim, 2014) and learning environment (Young, 2005; Alzubaidi et al., 2016), as strong motivating factors, promote student’s self-regulated learning.

#### Engagement and self-regulated learning

Zimmerman (1989) defines self-regulation as the extent to which learners are “metacognitively, motivationally and behaviorally active participants in their own learning process” (p. 329). Therefore, cognitive, affective, and behavioral engagements in learning are key to self-regulated learning. In addition, a number of studies have reported that engagement significantly contributes to self-regulation. Gaxiola-Romero et al. (2020) explored the connection between positive learning environments, academic engagement, and self-regulated learning. They found that a positive learning environment is linked to academic engagement, and academic engagement is related to self-regulated learning. Estévez et al. (2021) explored the contribution of cognitive, affective, and behavioral engagement to self-regulated learning. The result revealed that the highly engaged learners better controlled their time and study, were the most tactical in searching information, had less maladjusted regulatory actions, and gained the best scores.

### Materials and methods

#### Participants

In total, 419 undergraduate EAP students (178 male and 241 female) with an age range of 18–24 took part in this study. A stratified purposive sampling design was followed. We first organized the Iranian universities based on their ranking in light of the two strata of state-run and private sectors. Then, we purposefully selected potential candidates from each stratum. The majority of the participants were from universities in the capital (Tehran) and the rest came from northern, southern, western, eastern, and central regions in Iran. All the participants were taking the academic English courses at their universities and were practicing academic reading skill in the course of the study. These students were divided into several disciplinary groupings using Nesi and Gardner’s (2006) classification scheme:

1) Arts and Humanities: Arabic literature (36), philosophy (34), and history (32).
2) Life Sciences: medicine (42), psychology (33), and biology (32).
3) Physical Sciences: mechanical engineering (45), computer engineering (39), chemical engineering (9), railway engineering (8), and industrial engineering (5).
4) Social Sciences: law (54) and economy (50).

#### Instruments

Two sets of questionnaires were used to operationalize TE and L2MSS.

##### TE questionnaire

We modified the original questionnaire by Koskey et al. (2018) which was developed for use in science education. We followed the guidelines in the literature on scale development and made a series of modifications to the original instrument to enhance its relevance to L2 contexts and our EFL respondents. This involved conducting a series of semi-instructed interviews with EAP instructors and students and consulting the related literature on TE and academic reading skill in EAP. The first version of the newly developed questionnaire consisted of two parts. The first section comprised 32 statements about transformative engagement of students in academic reading skill on a five-point Likert scale from 1 (strongly disagree) to 5 (strongly agree). These items were reduced to 28 items as a result of piloting the instrument. The second part included items about demographic information (i.e., gender, age, self-reported level of English proficiency, grade, and field of study). Since the participants were Persian-speaking students, the questionnaire was translated into Farsi and then back-translated into English to ensure its readability and intelligibility. Cronbach’s alpha reliability analysis was performed on the items of TE and its related qualities using R-Studio (see Table 1). The obtained reliability coefficients (α) revealed that items of TE and its three qualities were highly reliable. The results of exploratory factor analysis will be reported in the results section.

**TABLE 1:** Reliability analysis of TE questionnaire and its related qualities.

Scale	Items	α
Motivated	1–11	0.87
Expansion of perception	12–18	0.83
Experiential value	19–28	0.86
TE questionnaire	1–28	0.93

##### L2 motivational self-system questionnaire

Another instrument used for measuring L2MSS was derived from a study conducted by Taguchi et al. (2009). In order to validate L2MSS, they developed a questionnaire for three contexts of Japan, China, and Iran. “All these versions were fine-tuned through extensive piloting in each of the three countries” (p. 74). They confirmed the validity of entire tripartite L2MSS using structural equation modeling. Moreover, Cronbach’s alpha analysis revealed the reliability of ideal L2 self (α = 0.79), ought-to L2 self (α = 0.75), and L2 learning experience (attitudes to learning English; α = 0.82) items for the context of Iran. The current study adopted the items developed for the context of Iran except for item 4 (which suited the contexts of Japan and China). This questionnaire consisted of two parts. The first part contains items measuring “ideal L2 self” and “ought-to L2 self” (items 1–13) which were randomly distributed and measured on a six-point Likert scale with 1 showing strongly disagree and 6 strongly agree. The second part included items assessing L2 learning experience (items 14–19) using a six-point Likert scale running from 1 (not at all) to 6 (very much). We translated and piloted the questionnaire with 20 EAP undergraduate students. It received good feedback, and no modification was needed. In order to assess the internal consistency of the questionnaire, the whole-scale and subscale reliability analyses were conducted by calculating Cronbach’s alpha coefficient in R-Studio program (see Table 2).

**TABLE 2:** Reliability analysis of L2 motivational self-system questionnaire.

Scale	Items	α
Ideal L2 self	1, 4, 6, 7, 9, 11, 13	0.88
Ought-to L2 self	2, 3, 5, 8, 10, 12	0.87
L2 learning experience	14, 15, 16, 17, 18, 19	0.85
L2 motivational self-system	1–19	0.86

The reliability coefficient (α) for L2MSS questionnaire and its subscales exceeded 0.80, which is considered high reliability.

#### Procedure

After getting permission from the universities and developing and piloting the questionnaires, a consent letter was sent to EAP instructors and students who were in the target universities to help us with data collection. Both questionnaires were then administered *via* e-mails to the instructors and delivered in person to 450 EAP students who were identified as potential respondents. Finally, the collected data were entered into statistical programs of R-Studio and IBM SPSS Statistics 24.0. Exploratory factor analysis was performed to determine the validity of TEQ. The contribution of L2MSS to TE was measured through simple linear regression. Moreover, the extent to which each subcomponent of L2MSS contributed to TE was measured through multiple regression. To determine whether there was any significant difference in the student’s motivation and engagement across disciplinary groups, MANOVA was run.

### Results

#### Instrument validation

##### TE questionnaire

The validity of TE questionnaire was examined through exploratory factor analysis (EFA) in SPSS program. To field test the modified questionnaire, we administered it to 171 respondents who were similar to the final target samples. This analysis was conducted through the principal components analysis method.

**FIGURE 1:**
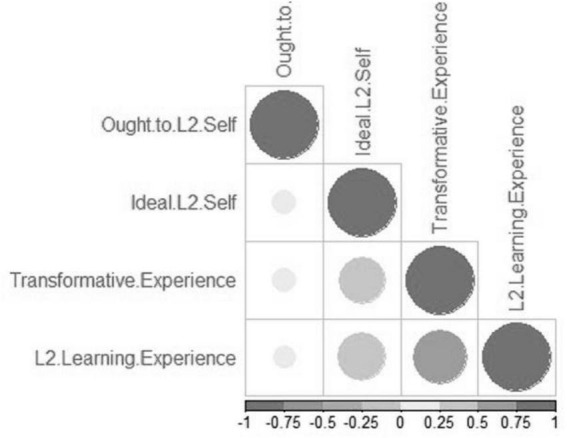
Regression plot of L2 motivational self-system subcomponents with transformative experience.

The result of KMO and Bartlett’s test verified the adequacy (KMO = 0.918) and factorability (*p* < 0.05) of the data. Given the results of component matrix (Table 1, Supplementary Appendix B) and the scree plot (Figure 1, Supplementary Appendix B), it seems that all items were related and the TE questionnaire enjoyed acceptable construct validity.

#### Descriptive findings

As presented in Table 3, data on transformative engagement showed that students were highly engaged in “experiential value,” which played a considerable role in enhancing the engagement of students in academic reading classes. This was followed by “motivated use” (MU) and “expansion of perception” (EP).

**TABLE 3:** Descriptive statistics for qualities of transformative experience.

Subcomponents	Mean	SD	Min	Max	Skewness	Kurtosis
Motivated use	35.9	7.8	11	55	−0.40	0.35
Expansion of perception	23.6	5.2	7	35	−0.52	0.64
Experiential value	38.2	6.3	17	50	−0.57	0.44

As shown in Table 4, the descriptive statistics of L2MSS subcomponents reveal that ideal L2 self, with the highest mean, was the most significant motivator for learning the English language for these university students. L2 learning experience with the mean value of 25.5 ranked as the second effective factor in motivating the students. The lowest mean belonged to ought-to L2 self which indicates this subcomponent had the least impact on L2 motivation of the students.

**TABLE 4:** Descriptive statistics for L2 motivational self-system subcomponents.

Subcomponents	Mean	SD	Min	Max	Skewness	Kurtosis
Ideal L2 self	**33.7**	**6.8**	7	**42**	**−0.98**	**0.79**
Ought-to L2 self	20.0	7.1	6	36	−0.10	−0.80
L2 learning experience	25.5	6.4	6	36	−0.45	−0.13

*RQ1: The relationship between L2 motivation and transformative engagement*

In order to check the normality of the data, the Shapiro–Wilk test was performed for both L2MSS and TE data. The test yielded the *p*-value of 0.000 (*p* < 0.05) for both TE and L2MSS data, indicating that these data were not normally distributed. Given that non-normality of data may result in bias and error in point estimates of Pearson’s product-moment coefficient (Bishara and Hittner, 2015), its major alternative, Spearman’s rank order correlation was used for exploring the relationship between L2MSS and TE. As shown in Table 5, Spearman’s correlation analysis has yielded a coefficient of 0.514, which suggests a significant relationship between students’ L2 motivation and transformative engagement in academic reading skill.

**TABLE 5:** Spearman’s correlation coefficient for transformative experience and L2 motivational self-system.

		Transformative experience	L2 motivational self-system
Transformative experience	Spearmancorrelation *p*-value	1	0.514 0.000
	*N*	419	419

*RQ2: Motivational factors as predictors of transformative engagement*

In order to explore the second research question, a simple linear regression was run between these two main variables using R-Studio. The obtained regression coefficient (β = 0.66) implies that TE is predicted to increase 0.66 when LMSS goes up by one unit.

In order to explore the extent to which subcomponents of L2MSS contribute to TE, a multiple regression analysis was run using R-Studio (see Table 6). The multiple regression analysis yielded the coefficient value of 1.40 (*p* < 0.05) for L2 learning experience variable, indicating that this subcomponent was the best predictor of the students‘ transformative engagement in reading skill. Ideal L2 self, with coefficient value of (β = 0.44; *p* < 0.00) appeared to have significantly contributed to TE. In contrast, the coefficient value of oughtto L2 self-variable was substantially lower than other motivational factors (β = 0.09) and not statistically significant. Hence, transformative engagement of students in reading skill may not be considerably affected by their family, friends, or people around them (see Figure 1).

**TABLE 6:** Multiple regression analysis of L2 motivational self-system subcomponents with transformative experience.

Variable	β	*P*-value	*T*-value	Std. Error
Ideal L2 self	0.44	0.00	4.23	0.10
Ought-to L2 self	0.09	0.28	1.07	0.08
L2 learning experience	1.40	0.00	12.43	0.11
*R*^2^ = 0.42				
*F*-value = 101.2

*RQ 3: Transformative engagement and L2 motivation across disciplines*

To examine any significant differences in student’s L2 motivation and transformative engagement across disciplinary groups, one-way multivariate analysis of variance (one-way MANOVA) was conducted for TE and L2MSS as dependent variables and disciplinary groups as independent variable (see Table 7).

**TABLE 7:** Descriptive statistics of transformative experience and L2 motivational self-system across disciplinary groups.

Dependent variable		*N*	Mean	SD
Transformative Eexperience	Arts and Humanities	102	95.54	18.24
	Life Sciences	107	99.42	15.96
	Physical Sciences	106	97.33	17.31
	Social Sciences	104	98.75	17.11
	Total	419	97.78	6.96
L2 motivational self-system	Arts and Humanities	102	76.36	16.16
	Life Sciences	107	82.19	12.38
	Physical Sciences	106	78.66	15.12
	Social Sciences	104	79.76	12.95
	Total	419	79.28	14.32

For TE, the mean value across the four disciplines ranges from 95 to 99 and for L2MSS from 76 to 82. The difference between the mean values is more considerable for L2MSS than for TE. Therefore, it can be presumed that disciplinary orientation influenced university students’ L2 motivation more than their transformative engagement in academic reading skill.

To measure the impact of disciplinary groups on TE and L2MSS, Test of Between-Subjects Effects was run. We found that disciplinary orientation did not significantly affect TE. However, L2MSS (*p* < 0.05) seems to have been significantly influenced by disciplinary grouping. To understand how disciplinary groups influence L2MSS, Tukey’s HSD *post hoc* test was run (Table 8).

**TABLE 8:** Post hoc test (Tukey’s HSD).

Dependent variable	(I)Disciplinary	(J)Disciplinary group	Mean differences (I - J)	Sig.
Transformative experience	Arts and Humanities	Life Sciences	−3.87	0.35
		Physical Sciences	−1.79	0.87
		Social Sciences	−3.21	0.52
	Life Sciences	Arts and Humanities	3.87	0.35
		Physical Sciences	2.08	0.80
		Social Sciences	0.66	0.99
	Physical Sciences	Arts and Humanities	1.79	0.87
		Life Sciences	−2.08	0.80
		Social Sciences	−1.42	0.93
	Social Sciences	Arts and Humanities	3.21	0.52
		Life Sciences	−0.66	0.99
		Physical Sciences	1.42	0.93
Motivational L2 self-system	Arts and Humanities	Life Sciences	−5.83*	0.01
		Physical Sciences	−2.30	0.64
		Social Sciences	−3.40	0.31
	Life Sciences	Arts and Humanities	5.83*	0.01
		Physical Sciences	3.52	0.27
		Social Sciences	2.42	0.60
	Physical Sciences	Arts and Humanities	2.30	0.64
		Life Sciences	−3.52	0.27
		Social Sciences	−1.09	0.94
	Social Sciences	Arts and Humanities	3.40	0.31
		Life Sciences	−2.42	0.60
		Physical Sciences	1.09	0.94

*The mean difference is significant at 0.05 level.

We notice that the level of L2 motivation was significantly different across Arts and Humanities and Life Sciences disciplines; hence, students were more motivated for learning the English language in Life Science compared to the latter group.

*RQ 4: Engagement inside and outside class*

**FIGURE 2:**
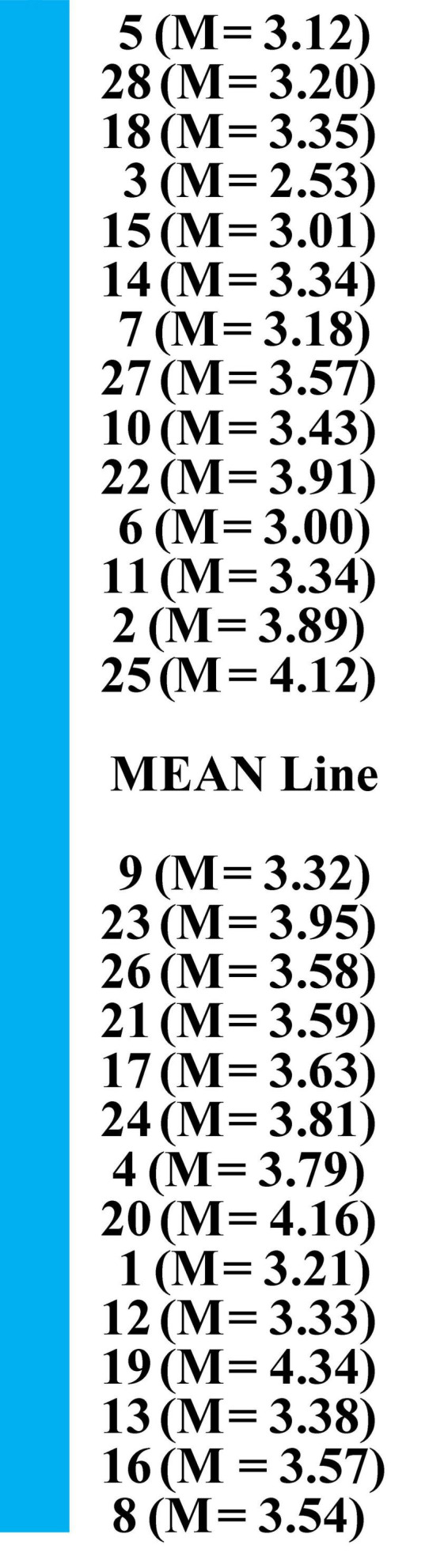
Continuum of in class and out—of—class engagement.

One of the functions of TEQ is to differentiate between students reporting “in-class from out of class engagement, with those agreeing to out-of-class engagement moving toward a higher degree of transformative experience” (Koskey et al., 2018, p. 116). Items of TE questionnaire were spread along a continuum (see Figure 2). Those items targeting in-class engagement are located at the lower end of the continuum and below the mean line. Directly below and above the mean line are items targeting students’ out-of-class engagement in academic reading. It is important to note that the mean value of items at the lower end of the continuum (in-class items) and the middle of the continuum (out-of-class items) was not significantly different.

## Discussion

Our descriptive findings indicated that EAP students were highly engaged in “experiential value,” “motivated use” (MU), and “expansion of perception” (EP), respectively. This implies that most students found academic reading skill useful in their lives and everyday experience, and a large number of students used academic reading skill strategies and knowledge in the contexts where it was not required (i.e., it was not their homework) especially outside class. Ideal L2 self, with the highest mean, was found to be the most significant motivator for university students in learning the English language. Hence, L2 motivation of the students mostly depended on how they imagined themselves using the English language in future. L2 learning experience was also an effective factor in motivating the students. It seems that learning environment of students, such as the impact of instructors, curriculum, peer group, and experience of success, plays a considerable impact on their L2 motivation. The lowest mean belonged to ought-to L2 self which implies that L2 motivation of the students was not highly under the influence of their family, friends, or people around them. This finding could be against the claim of several researchers (Taguchi et al., 2009; Kormos et al., 2011; Islam et al., 2013) arguing that ought-to self might appear more largely in Asian L2 contexts due to the considerable influence of family and other important people in Asian cultures.

Our findings further revealed that L2 motivation covaried with students’ level of engagement, thus signifying a positive relationship between L2MSS and in-class engagement (Asker, 2012). The present study, however, dealt with both in-class and out-of-class engagement. The significance of our findings lies in the positive contribution of L2MSS as an effective motivational factor in engaging students inside and outside class and overcoming distractors surrounding them. In this regard, Dornyei (2019b) argues that when students are engaged, they are necessarily powered by some motivation that guides their activities. Motivation is fundamental for engaging in TE (Alongi et al., 2016), and our findings provide empirical evidence for such interrelationship. Accordingly, motivational factors have proven to be successful players in passing over a combination of distractions, temptations, and alternatives around (Reyes and Johnston, 2018).

Our findings on the second research question revealed that L2 learning experience is the strongest predictor of motivated behaviors. L2 learning experience deals with learning environment of students, breaking down into facets such as school context, syllabus, teaching materials, learning tasks, peers, and teachers (Dornyei, 2019a). Therefore, it is inferred that atmosphere of the language class had the most effect on the transformative engagement of students in academic reading skill. In some way, it overlaps with Crawford (2018) and Pugh et al.’s (2010b) findings that peer group and teacher are, respectively, effective factors in the occurrence of TE.

Given that the ideal L2 self involves a desired self-image that one wishes to be in the future (You and Dörnyei, 2016), it can be concluded that the way students pictured themselves using the English language in the future had a significant effect on their transformative engagement in academic reading skill. Our results confirm studies such as Pugh (2004) and Pugh et al. (2010a) which reported that students’ imagined identity—including their possible selves—had a significant part in the occurrence of TE. That is, a positive and significant relationship between identity (including possible selves) and TE could be postulated (Girod and Wong, 2002; Pugh, 2004, cited in Pugh et al., 2010a). It should be noted that the aforementioned studies examined the role of science identity whereas the present study dealt with language identity.

As for the third research question, our findings reveal that disciplinary variations did not significantly affect TE. However, L2MSS seems to have been significantly influenced by disciplinary groups. Previous literature using L2MSS to assess the motivation across different disciplines (e.g., Martinović, 2018; Hosseini and Shokrpour, 2019) used different disciplinary grouping from the ones we used. We believe our disciplinary groupings are more empirically grounded (Nesi and Gardner, 2006). Our findings overlap partly with Martinović (2018) in which Humanities and Social Sciences students were motivated to almost the same level, whereas the results of the present study indicated that Life Sciences students were more motivated compared to Arts and Humanities ones.

Findings for research question four showed that EAP students were engaged in academic reading skill both inside and outside the class to more or less similar extent. This implies that successful language learning is contingent on two dimensions, namely, in-class and out-of-class engagement (Richards, 2015). In-class engagement is strongly related to effective learning (Fredricks et al., 2004) and language achievement (Dincer et al., 2019). Out-of-class engagement is associated with successful language development (Lai et al., 2015), English language awareness, and autonomy (Guo, 2011). Our findings lend support to learning contexts in which students appreciate academic reading both in- and out-of-class activities. They further highlight previous findings that reported positive effects of both types of academic reading on language achievement (Heng, 2014; Karabiyik, 2019).

Based on the findings of this study, we can argue that ideal L2 self, followed by L2 learning experience, would contribute to learner’s self-regulation in academic reading skills. Moreover, it is expected that TE, which deals with deeper levels of engagement, would facilitate self-regulated learning considerably. Indeed, out-of-class engagement, which is central to TE, allows learners to regulate and control their learning process independently. The important point here is that, in order for learners to put extra effort and time (i.e., get more engaged) into self-regulated learning, they must be motivated (Al-Otaibi, 2013). Given that this study found L2 learning experience as the strongest predictor of TE in academic reading, it is supposed that the learning environment takes a leadership role in students’ TE and in turn their self-regulation in academic reading skill. Young (2005) puts it best: “creating classroom environments that actively engage students both experientially and cognitively have the potential of stimulating the development of self-regulated learning” (p. 25).

## Conclusion

This study primarily attempted to explore potential relationships between L2 motivation and transformative engagement in academic reading among undergraduate EAP learners. The results revealed that transformative engagement of our target university students in academic reading skill was influenced by their L2 motivation to a significant extent and in a positive way. The implications can be for pedagogy, educational policy, and materials development. Teachers can foster engagement of their students in academic reading skill, both inside and outside the class, by promoting their motivation for learning a foreign language. Policy-makers are required to concentrate on L2 motivation of students when making educational programs for language courses to have better outcomes. Further, to get students more engaged in academic reading skill, materials and curricula need to be developed in a way that motivate learners for learning language.

Another objective of this study was to explore which motivational factors (i.e., subcomponents of L2MSS) are strong predictors of transformative engagement in academic reading skill. We found that L2 learning experience and ideal L2 self played the most important role in the engagement of students; however, ought-to L2 self had a marginal part in this regard. Given that L2 learning experience and ideal L2 self relate to learning environment and one’s self-image, respectively, teachers in their attempts to engage student’s in academic reading skill can improve the atmosphere of class in different ways and foster students imagination for using foreign language in the future. Authorities can make the educational environments more interesting and appropriate for students and train good teachers in terms of psychological literacy to promote students’ self-image. Teaching material, as one aspect of L2 learning experience (Dornyei, 2019a), can be effective in engaging students. Thus, it is suggested that materials are motivating and contain subjects and ideas that provoke student’s self-imagination.

The current research also aimed to determine whether L2 motivation and engagement of students differ across the four disciplinary groups. Life Sciences students were the most, and Arts and Humanities ones were the least motivated for learning English. However, the engagement of the students was not substantially different from each other across disciplines. Teachers, educational authorities, and materials and curricula developers need to consider disciplinary variations and take measures for motivating students.

The differences between students’ engagement inside and outside class demonstrated that students were engaged in academic reading skill in and beyond class to roughly the same extent. It is suggested that teachers, educational authorities, and material and curricula developers pay attention to both in- and out-of-class contexts to engage students successfully in academic reading skill development.

The present study took a number of subject areas as the sample of each disciplinary group. This investigation was delimited to EAP undergraduate students. Considering other grades (e.g., high school or postgraduate level) and different learning contexts (e.g., private institutes or schools) is recommended for future research. Using mixed-method research and taking advantage of different research instruments, such as interviews, diary writing, and observation, can be useful in triangulating the relationship between motivation and transformative experience.

The findings of this study have fruitful implications for pedagogy, educational policy, and material development. Given the L2 learning experience was found as the strongest predictor of the student’s engagement, teachers can foster engagement of their students in academic reading skill by making the learning environment more interesting. The learning environment incorporates many elements, such as teachers, peers, classroom, educational facilities, and materials. Given the reported relationships, teachers can also promote student’s self-regulation in reading by increasing their L2 motivation and TE in academic reading. Therefore, students can better manage reading process and comprehend different types of texts independently. The self-regulated students tend to use the appropriate strategies for comprehending and gaining information from different texts and better manage their time when reading. Moreover, policy-makers are suggested to concentrate on L2 motivation of students when making educational programs for language courses to have a better outcome. In addition, to get students more engaged in academic reading skill, materials and curricula are better to be developed in a way that motivate learners for leaning language.

This study employed a quantitative approach to address the research questions. Adopting a mixed-methods design in future research can help depict not only the overall picture but more nuanced aspects of transformative engagement in reading performance of readers across disciplines. Further, performing structural equation modeling (SEM) to gain a deeper understanding of the direction of the relationships between and among variables for model building purposes about TE integers would be beneficial.

## Data availability statement

The original contributions presented in this study are included in the article/Supplementary material, further inquiries can be directed to the corresponding author.

## Ethics statement

The studies involving human participants were reviewed and approved by Iran University of Science and Technology. The patients/participants provided their written informed consent to participate in this study.

## Author contributions

EA supervised the project from inception to the end and multiple revisions of the draft. MA co supervised the project from inception to the end and multiple revisions of the draft. MZ conducted the project and produced the initial report. All authors contributed to the article and approved the submitted version.
