# Supplementary material for: The relationship between L2 motivation and transformative engagement in academic reading among EAP learners: Implications for reading self-regulation
Source: Front Psychol. 2022 Sep 29;13:944650. doi: 10.3389/fpsyg.2022.944650 (PMC9558267; doi:10.3389/fpsyg.2022.944650)
Supplement: Supplementary file 1 [file Data_Sheet_1.pdf]

Appendix A

Part 1: Transformative experience in reading questionnaire (Adapted from Koskey et al, 2018).

Transformative Experience items	Strongly disagree	disagree	neutral	agree	Strongly agree
1- During my English class, I talk about reading topics with other students or the teacher.					
2- I think about learning English, outside the classroom, by reading different texts including English books, news, etc.					
3- After reading class, I talk about the reading topics with my friends and family.					
4- During my English class, I think about different topics appeared in lessons.					
5- I enjoy talking about interesting English books with classmates.					
6- Outside the class, I think about different topics appeared in lessons.					
7- Outside the class, I find myself thinking about different things I have learned in reading class (e. g. vocabulary, grammar, reading strategies, etc.).					
8- During my English class, I apply the reading strategies the teacher taught.					
9- Outside the class, I apply the reading strategies the teacher taught.					
10- I apply the stuff I have learned about reading English even when I don't have to.					
11- I seek out opportunities to apply my knowledge of reading outside the class.					
12- When I read different English texts (e. g. books, news, social media, websites, etc.) I think about it in terms of factors of reading (e. g. vocabulary, grammar, reading strategies, etc.)					
13- When I`m working on a class assignment about reading comprehension, I tend to think about it in terms of factors of reading (e. g. vocabulary, grammar, reading strategies, etc.).					
14- If I read a really interesting text outside of class, then I think about it in terms of factors of reading (e. g. vocabulary, grammar, reading strategies, etc.).					
15- When I read English texts ((e. g. books, news, social media, websites, etc.), I can`t help					

but think about it in terms of factors of reading (e. g. vocabulary, grammar, reading strategies, etc.).					
16- During my English class I notice samples of English reading texts.					
17- I notice samples of English reading texts outside the class (e. g. English news, social media, websites, etc.).					
18- I look for samples of English reading texts outside the class (e. g. English news, social media, websites, etc.).					
19- Learning about reading is useful in my field of study.					
20- Knowledge of reading helps to increase my background knowledge.					
21- Reading is daily activity which is extremely useful in my current life outside the class.					
22- I find that knowledge of reading keeps me updated.					
23- Knowledge of reading makes English books more interesting.					
24- In my English class I find it interesting to learn new things through reading.					
25- I think reading is an interesting topic.					
26- I find it interesting in class when we talk about reading topics.					
27- I`m interested when outside the class I read materials related to the reading lessons I have learned.					
28- I find it exciting to think outside of class about reading lessons I have learned.					

Part 2:

Gender: male ☐ Female ☐

Age: ………………

Level of English language proficiency:

Beginner ☐ lower-intermediate ☐ intermediate ☐ upper-intermediate ☐ advanced ☐

Grade:

Associate degree ☐ Bachelor`s degree ☐ Master`s degree ☐ Doctoral degree ☐

Field of study: ……………………….

Appendix B

Table 1. Factor Matrix on dimensions of TE

Item		Component Loading 1
Item 11	**MU**	0.780
Item 26	**EV**	0.766
Item 28	**EV**	0.717
Item 7	**MU**	0.675
Item 27	**EV**	0.674
Item 17	**EP**	0.671
Item 24	**EV**	0.660
Item 10	**MU**	0.659
Item 9	**MU**	0.644
Item 6	**MU**	0.640
Item 23	**EV**	0.635
Item 4	**MU**	0.630
Item 25	**EV**	0.626
Item 2	**MU**	0.611
Item 5	**MU**	0.602
Item 13	**EP**	0.602
Item 14	**EP**	0.601
Item 12	**EP**	0.601
Item 15	**EP**	0.598
Item 22	**EV**	0.586
Item 18	**EP**	0.563
Item 1	**MU**	0.560
Item 20	**EV**	0.559
Item 16	**EP**	0.558
Item 3	**MU**	0.550
Item 21	**EV**	0.537
Item 19	**EV**	0.527
Item 8	**MU**	0.509

*MU = Motivated Use
EV = Experiential Value
EP = Expansion of Perception

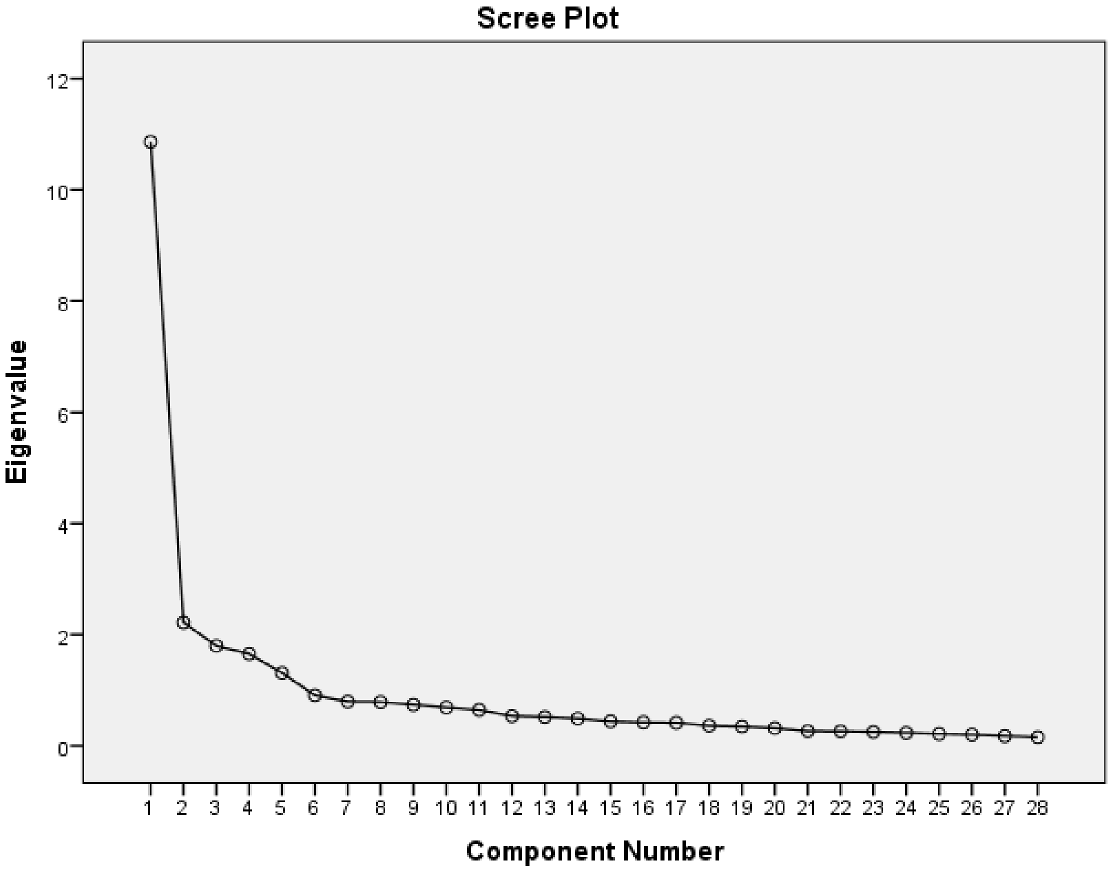

Figure 1. Scree Plot of Exploratory Factor Analysis
